# Inner Speech and Clarity of Self-Concept in Thought Disorder and Auditory-Verbal Hallucinations

**DOI:** 10.1097/NMD.0000000000000584

**Published:** 2016-11-30

**Authors:** Paulo de Sousa, William Sellwood, Amy Spray, Charles Fernyhough, Richard P. Bentall

**Affiliations:** *Institute of Psychology, Health and Society, University of Liverpool, Liverpool; †Division of Health Research, Faculty of Health and Medicine, Lancaster University, Lancaster; ‡School of Psychology, University of Liverpool, Liverpool; and §Department of Psychology, Durham University, Durham, UK.

**Keywords:** Thought disorder, hallucinations, inner speech, self-concept, psychosis

## Abstract

Eighty patients and thirty controls were interviewed using one interview that promoted personal disclosure and another about everyday topics. Speech was scored using the Thought, Language and Communication scale (TLC). All participants completed the Self-Concept Clarity Scale (SCCS) and the Varieties of Inner Speech Questionnaire (VISQ). Patients scored lower than comparisons on the SCCS. Low scores were associated the disorganized dimension of TD. Patients also scored significantly higher on condensed and other people in inner speech, but not on dialogical or evaluative inner speech. The poverty of speech dimension of TD was associated with less dialogical inner speech, other people in inner speech, and less evaluative inner speech. Hallucinations were significantly associated with more other people in inner speech and evaluative inner speech. Clarity of self-concept and qualities of inner speech are differentially associated with dimensions of TD. The findings also support inner speech models of hallucinations.

“Truth is not born nor is it to be found inside the head of an individual person; it is born between people collectively searching for truth, in the process of their dialogic interaction.” (Bakhtin, 1929, p.110).

Thought disorder (TD) refers to a multidimensional and transdiagnostic cluster of cognitive, linguistic, and communication disturbances that compromise the sharing of meaning during conversation (Andreasen, 1986; Cuesta and Peralta, 1999) and that are highly prevalent in schizophrenia with some estimates reaching 91% (Roche et al., 2015). TD has been found to be a significant predictor of conversion into psychosis in high-risk populations (Bearden et al., 2011; Cannon et al., 2008; Ott et al., 2002) and has been associated with a range of adverse outcomes such as psychotic relapse (Wilcox, 1990), poorer occupational (Racenstein et al., 1999) and social functioning (Bowie et al., 2011; Bowie and Harvey, 2008), and poorer quality of life (Tan et al., 2014). Despite its clinical relevance, TD is still a poorly understood phenomenon and evidence-based therapeutic approaches are nearly nonexistent (Beck et al., 2009).

A variety of theories have been produced to explain TD, from poor executive ability (Kerns and Berenbaum, 2002; McGrath, 1991; McGrath et al., 1997; Stirling et al., 2006), disorganization of semantic networks (Goldberg and Weinberger, 2000; Goldberg et al., 1998), a hyperpriming effect in semantic memory (Pomarol-Clotet et al., 2008; Spitzer, 1997) to deficits at the level of context representation (Cohen and Servan-Schreiber, 1992; Roesch-Ely et al., 2010). Neurobiological correlates include decreased gray matter volume in the left posterior superior temporal gyrus, which has also been associated with auditory verbal hallucinations (Shenton et al., 1992; Subotnik et al., 2003; Vita et al., 1995); decreased activity in the inferior frontal, cingulate, and left superior temporal cortex while patients are asked to describe ambiguous pictures (McGuire et al., 1998); and abnormal dorsolateral prefrontal activity during functional magnetic resonance imaging studies (Goghari et al., 2010; Roesch-Ely et al., 2010).

It has been argued that the perceived unintelligibility of TD (Beck et al., 2009; Bentall, 2003) may in fact reflect the intermingling of decontextualized personal concerns and worries (Harrow et al., 1983; Lanin-Kettering and Harrow, 1985) coupled with a loss of perspective (Harrow et al., 2000) or poor theory of mind (Frith, 1992; Hardy-Baylé et al., 2003) making it difficult for the speaker to adjust their speech according to the needs of the listener. TD has been observed to become more pronounced when patients are asked to disclose negative autobiographical memories (Shimkunas, 1972; Tai et al., 2004) or affect-laden material (Docherty et al., 1994a, 1994b; Docherty, 1996; Docherty et al., 1998; Mohagheghi et al., 2013).

## 

### TD as Disruption of Inner Dialogue

One outstanding question concerns whether the organization of the self-construct and the corresponding production of a self-narrative impacts upon patients’ ability to engage in patterned and organized dialogues with others.

A useful theoretical framework within which it is possible to consider this question is Dialogical Self Theory (DST, Hermans et al., 1992), which draws on philosophy (James, 1983; Nietzsche, 1997) and literary scholarship (Bakhtin, 1929) in understanding the self as an assembly or *society* of coexisting internal and external self-positions (or *I*-positions), which are hierarchically arranged, and in which the self is the dialogical narrator (Hermans et al., 1992; Lysaker and Lysaker, 2010). Internal self-positions refer to our different representations of our identity and social roles (*e.g.*, *I*-as a husband or *I*-as a jazz lover) whereas external self-positions are the people that populate our worlds and to whom we are affectively bonded (*e.g.*, my friend who also loves jazz). A coherent sense of self is dependent on the communication or dialogue between the different self-positions that can be either complementary or contradictory. Internal coherence is achieved and sustained through the dynamic generated by this inner dialogue and by outer dialogue with others.

It has been argued that the disturbances of self-experience documented in psychosis, such as diminished sense of identity and agency (Frith, 1992; Sass, 2014), are related to a collapse of the dialogue of self-positions within the individual and between the individual and other people (Lysaker and Lysaker, 2001, 2002, 2005). Lysaker and Lysaker (2002) have proposed three types of potential disruptions to dialogue: (1) a suspension of inner and outer dialogue, *e.g.*, poverty of speech; (2) lack of a dialogical hierarchy and socially validated coherence, *e.g.*, positive TD (Lysaker and Lysaker, 2006); and (3) the compromise of dialogue rigid self-position, *e.g.*, delusional beliefs.

Some studies have examined the self-concept in patients diagnosed with schizophrenia. For example, Cicero and colleagues (Cicero et al., 2013) reported that the interaction between poor self-concept clarity and aberrant salience was a significant predictor of psychotic-like experiences whereas a larger body of research has suggested a more specific association between negative self-concept and paranoia (Tiernan et al., 2014). According to DST, however, TD should relate to a lack of self-concept clarity resulting from the emergence of a cacophonous self.

It should also be possible to trace disruptions to the inner dialogue through the phenomenon of inner speech, the internal flow of verbal thought that characterizes many people’s conscious experience (Fernyhough, 2013). According to the Dialogic Thinking Model (DTM, Fernyhough, 1996, 2009), inner speech has a dialogic character, which reflects its developmental origins in social exchanges (Vygotsky, 1934). Inner speech has also been proposed to exist in different forms corresponding to different levels of expansion and dialogicity (Fernyhough, 2004), a proposal that has received empirical support (Alderson-Day et al., 2014; McCarthy-Jones and Fernyhough, 2011). Fernyhough (2004) proposed that auditory verbal hallucinations (AVHs) in psychosis might be caused by a disruption to the process whereby condensed inner dialogue is expanded into a more overt internal dialogue between differing points of view. However, this hypothesis was not supported in a study by Langdon and colleagues (2009), which showed a nonsignificant trend towards reduced dialogicity in inner speech in psychotic patients with AVHs but no significant differences in inner speech quality compared with healthy controls.

### Aims of the Study

In the present study, we wanted to investigate if lack of self-concept clarity was more prevalent in patients diagnosed with psychotic-spectrum disorders and, if so, whether this lack of clarity was significantly associated with TD during an interview designed to promote personal disclosure. In line with predictions from both DST and the DTM, we also wanted to test whether patients diagnosed with psychotic-spectrum disorders reported experiencing less dialogic inner speech and if this might be associated with TD. At a more exploratory level, we wanted to test how these variables related to the different dimensions of TD. To test the specificity of these hypotheses, we decided to control for other psychotic experiences (*e.g.*, hallucinations and delusions) given that self-concept has been found to be an important variable in paranoia (Tiernan et al., 2014) and inner speech has been conceptualized as an important aspect of auditory verbal hallucinations (Alderson-Day et al., 2014; Bentall, 1990; Fernyhough, 2004).

A secondary purpose of the present study was to also test hypotheses about the relationship between inner speech and hallucinations, given that previous studies with nonclinical samples have found associations between both other people in inner speech and motivational/evaluative inner speech and proneness to auditory hallucinations (Alderson-Day et al., 2014; McCarthy-Jones and Fernyhough, 2011) whereas clinical studies have not (Langdon et al., 2009).

## METHODS

### Participants

As part of a wider study of the determinants of TD, we recruited 80 clinical participants who were experiencing psychotic symptoms (see Table 1) from local mental health sites across the North West of England. The recruitment targeted 18–65-year-olds with a psychotic-spectrum disorder as primary diagnosis defined as schizophrenia, schizoaffective according to ICD-10 (World Health Organization, 2004), or, in the case of early intervention services (where there was a reluctance to use formal diagnoses), “other psychosis.” The presence of psychotic symptoms was confirmed using data from the PANSS (see below) and clinical history.

**TABLE 1:**
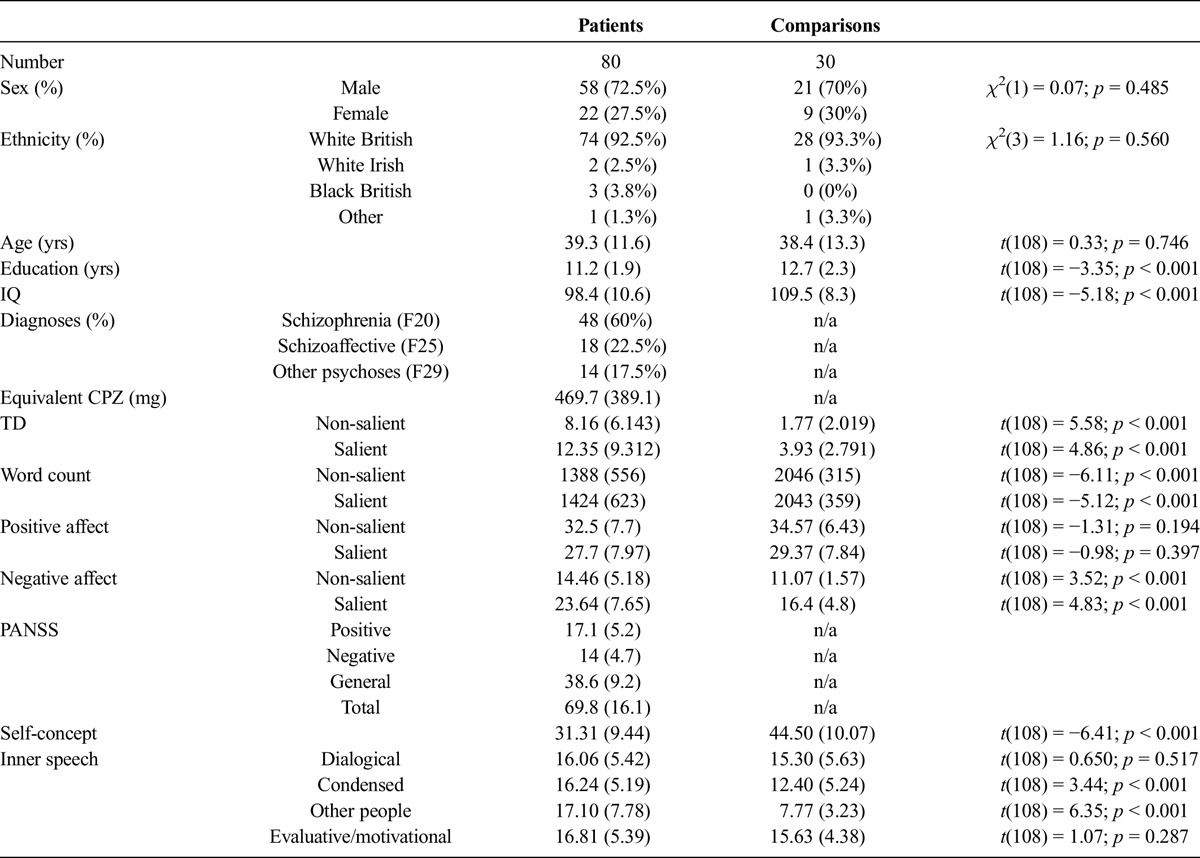
Group Data on Clinical, Demographic, and Psychological Variables

We excluded participants who lacked capacity for informed consent, whose first language was not English, and individuals with diagnosed learning difficulties, recent substance abuse, or a history of neurological disorders. Antipsychotic medications were converted to chlorpromazine equivalents as per agreed conventions (Woods, 2003). For comparison purposes, 30 participants were recruited through local advertisements in the community and screened for psychotic symptoms with the Psychosis Screening Questionnaire (PSQ, Bebbington and Nayani, 1995). An attempt was made to select participants who were approximately comparable with our participants in the clinical group on variables such as gender, age, and ethnicity.

### Measures

#### Psychotic Symptoms

Psychotic symptoms were measured using the *Positive and Negative Syndromes Scale* (PANSS, Kay et al., 1987). The PANSS is a widely used clinical interview that measures 30 symptoms, comprising a positive symptom subscale, a negative symptom subscale, and a general psychopathology subscale. Each item is scored from 1 to 7 with the higher score indicating increased severity. The scale has been found to have good psychometric properties (Kay et al., 1987).

#### Quick Test

Verbal intelligence was measured using the Ammons Quick test (QT, Ammons and Ammons, 1962), an untimed picture vocabulary test. The participant is presented with four pictures of different situations and is asked to identify 50 progressively difficult words by simply pointing to the appropriate card where the word referent can be found and the number of words correctly identified yields the total score. The QT has been extensively used in clinical studies and correlates with WAIS scores (Lezak, 2004).

#### Interviews

Speech samples were gathered from all participants using two interview protocols previously developed to elicit TD (Haddock et al., 1995; Tai et al., 2004). The protocols elicited speech samples relating to emotionally laden (salient interview) and neutral (non-salient interview) topics, given the evidence that participants diagnosed with psychosis show more TD when asked to talk about emotional material (Docherty et al., 1994a; Docherty, 2005; Shimkunas, 1972). The salient interview involved 15 questions that promoted self-disclosure by asking for negative autobiographical memories, whereas the non-salient interview included 15 questions that did not promote self-disclosure (see Appendix 1 for interview items).

#### Affect

Affect was measured with the Positive and Negative Affect Scale (PANAS; Watson et al., 1988), which assesses positive and negative mood using 20 words (*e.g.*, excited, jittery, nervous) rated by participants according to how they felt during the interview using a 5-point scale. The measure has good psychometric qualities (Watson et al., 1988). Means and standard deviations for both groups across interviews can be seen in Table 1.

#### TD

The speech samples were rated using the Scale for the Assessment of Thought, Language and Communication (TLC, Andreasen, 1986), a widely used scale that provides definitions and scores for 18 different items of TD (see Table 2) and has been supported by researchers in the field (Roche et al., 2015). The different categories of TD are rated on a scale of severity ranging from 0 to 4 or 0 to 3. The global rating is achieved by summing the scores of the different subscales. The scale can be applied to any speech samples and has been shown to have good psychometric properties (Andreasen, 1979a, 1986).

**TABLE 2:**
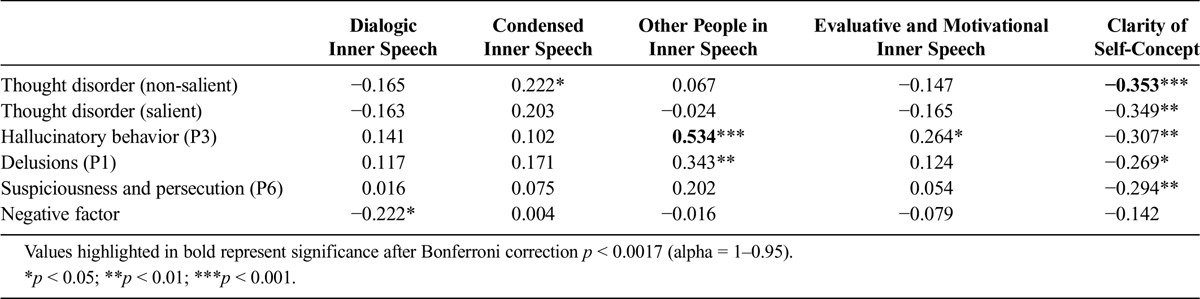
Bivariate Correlations Between TD, Hallucinations, Delusions, Suspiciousness/Persecution, Negative Symptoms, Four Dimensions of Inner Speech, and Clarity of Self-Concept (Clinical Group Only)

#### Self-Concept Clarity

The Self-Concept Clarity Scale (SCCS, Campbell et al., 1996) is a self-report questionnaire of 12 items that measures the extent to which beliefs about self are clearly defined, stable, and consistent. All the items are presented as sentences (*e.g.*, “In general, I have a clear sense of who I am and what I am.”) and the participant has to choose on a scale of 5 (1 = strongly disagree to 5 = strongly agree) how that statement reflects their own perception about their self. Psychometric properties of the scale have been found to be very good (Campbell et al., 1996), and the scale has been used in psychosis research (Cicero et al., 2013). In the present sample, the Cronbach alpha coefficient was 0.92.

#### Quality of Inner Speech

The Varieties of Inner Speech Questionnaire (VISQ, McCarthy-Jones and Fernyhough, 2011a) is a self-report questionnaire designed to assess the phenomenological properties of inner speech. The VISQ has 18 items presented in the form of sentences (*e.g.*, “I talk back and forward to myself in my mind about things.”), which the participant has to endorse using a 6-point Likert scale (ranging from 6 = “Certainly applies to me.” to 1 = “Certainly does not apply to me.”). The questionnaire is composed of subscales, namely (1) dialogic inner speech, (2) condensed inner speech, (3) other people in inner speech, and (4) evaluative and motivational inner speech. The scale has been found to have good psychometric properties. Cronbach alphas for the current sample were dialogical inner speech, *α* = 0.85; condensed inner speech, *α* = 0.67; other people in inner speech, *α* = 0.90; and motivational/evaluative inner speech, *α* = 0.81.

### Procedure

The present study was part of a larger research project on the social, cognitive, and affective predictors of TD approved by the UK National Research Ethics Service (NRES).

All participants in the study were seen twice on different days. The interval between the two sessions was in most cases a few days and never more than 1 week to prevent “carry-over” effects. Participants in the clinical group were interviewed with the PANSS (Kay et al., 1987) whereas controls were screened with the PSQ (Bebbington and Nayani, 1995). After these assessments, participants completed the QT before being interviewed.

Each participant was interviewed using the salient and non-salient interviews (Haddock et al., 1995; Tai et al., 2004) in a randomly counterbalanced order across the two sessions. Interviews lasted approximately 15 minutes on average, providing authors with 30 minutes of speech per participant. In the second session, participants were requested to complete both the VISQ and the SCC questionnaires. The speech samples were recorded with a digital voice recorder (Olympus VN711 PC 2GB) and later transcribed by the first author and a professional transcriber, before being coded independently by PS and AS using the TLC.

### Statistical Analysis

Statistical analyses were carried out on IBM SPSS Statistics (21.0.0). We used *χ*^2^, *t*-tests, and 2 × 2 mixed ANOVA to compare groups on both demographic and clinical variables. To further explore relationships between variables, we conducted bivariate and partial correlations (applying Bonferroni corrections to adjust for multiple comparisons) and two-staged linear regressions. Finally, to determine the different dimensions of TD, we conducted a factor analysis using an unweighted least squares method with varimax rotation. The cut-off criterion for the factors was *eigenvalues* greater than 1.

## RESULTS

### Demographic and Clinical Variables

The descriptive statistics for the demographic and clinical variables can be found elsewhere (De Sousa et al., 2015) and in Table 1. Briefly, the groups did not differ significantly on variables such as gender, age, or ethnicity. The only significant differences were on years of education with our comparisons reporting more years of education.

To test inter-rater reliability for the PANSS scores, two trained raters have independently coded the interviews of 10% of the clinical sample. Intraclass correlations (ICC) values were calculated for the positive, negative, and general factors, separately. All the positive and general symptoms were found to be above acceptable levels of reliability (>0.7) with the negative factor achieving the lowest level of agreement. The means and standard deviations of the PANSS factors approximate to the values reported in other patient studies (Kay et al., 1987).

The coding of the speech samples was preceded by the careful reading of the TLC and relevant papers (Andreasen and Grove, 1986; Andreasen, 1979a, 1979b, 1986) and by practice sessions. After this training period, PS and AS independently coded 10% (22) of the speech samples to test inter-rater reliability. It was not possible to calculate a Kappa value for items such as neologisms or clanging as they were found to be very infrequent. For the remaining items, all Kappa values were of substantial magnitude with tangentiality achieving the highest level of agreement (*k* = 0.82) and self-reference the lowest (*k* = 0.62).

As detailed elsewhere (De Sousa et al., 2016), our clinical group exhibited more TD than our comparison group, especially during the salient interview. They also reported more negative affect in both interviews, and, as expected, this difference was more pronounced in the salient interview.

### Group Differences on Psychological Measures

The correlations between SCCS and the VISQ subscales were, respectively, dialogical inner speech, *r* = −0.237, *p* = 0.013; condensed inner speech, *r* = −0.329, *p* < 0.001; other people in inner speech, *r* = −0.504, *p* < 0.001; and evaluative/motivational inner speech, *r* = −0.293, *p* = 0.003. Hence, lack of self-concept clarity was associated with low scores on all of the inner speech dimensions.

Means and standard deviations on the SCCS and the VISQ subscales for patients and comparisons, together with significance tests, are presented in Table 1. As expected, the patients scored significantly lower than the comparisons on the SCCS. Group differences were also observed for condensed inner speech and other people in inner speech but not on dialogic or evaluative/motivational inner speech.

Both PANSS anxiety (*r* = −0.384; *p* < 0.001) and PANSS depression (*r* = −0.223; *p* = 0.046) were found to be associated with SCC. Regarding the inner speech variables, the only significant association was between PANSS anxiety and “other people in inner speech” factor (*r* = 0.235; *p* = 0.036). Because anxiety and depression might plausibly inhibit speech production, we also explored relationships between these symptoms and TLC poverty of speech scores. The correlations with PANSS anxiety were nonsignificant (neutral: *r* = 0.138; *p* = 0.221; salient: *r* = 0.133; *p* = 0.241). However, PANSS depression scores were marginally associated with TLC poverty of speech in the salient interview (*r* = 0.235; *p* = 0.036) but not in the neutral interview (*r* = 0.194; *p* = 0.085).

### Clarity of Self-Concept and Individual Psychotic Experiences

Table 2 shows the bivariate correlations between self-concept clarity and the different psychotic symptoms for our clinical sample. Clarity of self-concept negatively correlated with all symptoms with the exception of the negative PANSS subscale. After applying Bonferroni corrections for multiple comparisons, only the correlation between TD in the neutral interview and clarity of self-concept remained significant. However, our prediction was that self-concept clarity would be significantly associated with TD scores even after controlling for comorbid symptoms. To test this prediction, we ran two two-stage linear regressions on the data from our clinical participants.

In the first regression model, we used the TD score from the non-salient interview as the dependent variable and, to control for co-occurring symptoms, we entered PANSS scores for hallucinatory behavior (P3), delusions (P1), and suspiciousness/persecution (P6) in the first stage. This initial model was significant, *F*[3, 76] = 5.19, *p =* 0.003, *R*^2^_adjusted_ = 0.137. Adding clarity of self-concept led to a significant improvement in the model, *F*_change_[1, 75] = 5.51, *p* = 0.022, leading to a significant final model, *F*[4, 75] = 5.5, *p* = 0.001, *R*^2^_adjusted_ = 0.186, in which clarity of self-concept was a significant predictor of TD in the non-salient interview (*b* = −0.261, *p* = 0.022). However, delusions also remained a significant predictor (*b* = 0.322, *p* = 0.01). After repeating the analysis with PANSS depression and anxiety scores added to the covariate list in the first step, the results remained substantially unchanged, with self-concept clarity (*b* = −0.252, *p* = 0.034) and delusions (*b* = 0.339, *p* = 0.008) still predicting TD.

We repeated the same procedure with TD scores from the salient interview as the dependent variable. Again the first model was significant, *F*[3, 76] = 4.25, *p =* 0.008, *R*^2^_adjusted_ = 0.110. Adding clarity of self-concept led to a significant improvement in the model, *F*_change_[1, 75] = 6.07, *p* = 0.016, leading to a significant final model, *F*[4, 75] = 4.91, *p* = 0.001, *R*^2^_adjusted_ = 0.165, in which clarity of self-concept was a significant predictor (*b* = −0.277, *p* = 0.016); this time the comorbid symptoms were not significant predictors. Repeating the analysis with the addition of depression and anxiety added to the comorbid symptoms led to very similar results, with only self-concept clarity predicting TD (*b* = −0.271, *p* = 0.024).

### Inner Speech and Individual Psychotic Experiences

To test the relations between the different psychotic symptoms and the four dimensions of inner speech, we conducted exploratory bivariate correlations for our clinical sample (see Table 2). The only significant correlations were between other people in inner speech and hallucinations, delusions and the positive PANSS factor, and between hallucinations and evaluative and motivational speech. Condensed inner speech was correlated with TD only in the non-salient interview and dialogic inner speech was only marginally correlated with negative symptoms (*p* = 0.047). After applying Bonferroni corrections for multiple comparisons, the only correlation that remained significant was between hallucinations and other people in inner speech.

In the light of the apparent strong correlation between delusions and other people in inner speech in the uncorrected correlations, and given that hallucinations and delusions often co-occur, we ran partial correlations between other people inner speech and each symptom, controlling for the other. The association with hallucinations remained strong (*r* = 0.47, *p* < 0.001) whereas the association with delusions was nonsignificant (*r* = 0.19, *p* = 0.09).

### Dimensions of TD, Inner Speech, and Clarity of Self-Concept

Because TD is a multidimensional construct (Andreasen and Grove, 1986; Berenbaum and Barch, 1995; Harvey et al., 1984; Peralta et al., 1992; Solovay et al., 1986), we decided to test how our psychological measures related to the different dimensions of TD. To extract factors from the TLC, we conducted a factor analysis using the 18 TLC scores from the salient interview of our clinical group and the unweighted least squares (ULS) method with varimax rotation (given that the TLC scores did not meet the criteria for the maximum likelihood). Our factor analysis produced six factors with *eigenvalues* greater than 1, similar to previous findings (Cuesta and Peralta, 1999). These six factors explained 69.64% of the total variance and were interpreted as *disorganized* (derailment, incoherence, illogicality, clanging, word approximations, circumstantiality, loss of goal, perseveration, and self-reference), *linguistic* (neologisms and stilted speech), *attentional* (pressure of speech and distractible speech), *poverty* (poverty of speech and tangentiality), *emptiness* (poverty of the content of speech and echolalia), and finally *blocking factor* (blocking). Table 3 shows the partial correlations between the different TD factors and clarity of self-concept after controlling for hallucinations, delusions, and suspiciousness/persecution.

**TABLE 3:**
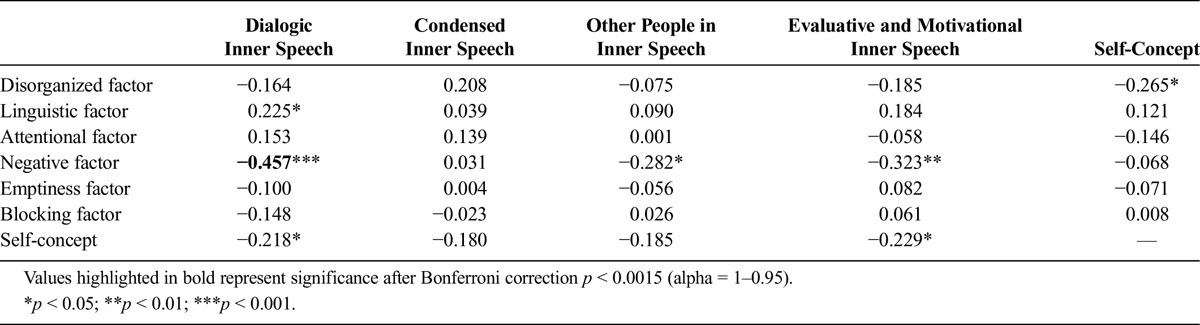
Partial Correlations Between TD Factors, Inner Speech Factors, and Clarity of Self-Concept After Controlling for Hallucinations, Delusions, and Suspiciousness/Persecution (Clinical Group Only)

To test the associations between the TD factors and the inner speech factors, we conducted exploratory partial correlations controlling for PANSS scores for hallucinatory behavior (P3), delusions (P1), and suspiciousness/persecution (P6). Table 3 shows that the negative TD factor was substantially and negatively correlated with self-reported dialogic inner speech but also with other people in inner speech and evaluative and motivational inner speech. The only other significant correlation was between the linguistic TD factor and dialogic inner speech; however, this was a positive correlation and marginally significant (*p* = 0.049). After applying Bonferroni corrections for multiple comparisons, the only association that remained significant was the negative correlation between the negative TD factor and self-reported dialogical speech.

## DISCUSSION

The primary goal of the present study was to explore associations between clarity of self-concept (the extent to which beliefs about self are stable across time, consistent and clearly defined; Campbell et al., 1996), self-reported inner speech, and TD. Interestingly, poor self-concept clarity was modestly associated with low scores on all of the inner speech dimensions. This finding is consistent with the suggestion by some theorists that the quality of inner speech contributes to self-knowledge and hence the coherence of the self-concept (Morin and Everett, 1990; Morin, 2007). Nonetheless, specific associations were found between self-concept clarity on the one hand and inner speech on the other and different psychotic symptoms.

As expected, our patients showed diminished clarity of self-concept. Although poor clarity of self-concept was associated with all psychotic experiences with the exception of the negative symptoms, our regression analyses revealed that it was a significant predictor of TD scores in both the salient and non-salient interviews, even after controlling for the other symptoms. Moreover, when specific TD factors were considered and co-occurring symptoms controlled for, lack of self-clarity was specifically associated with the disorganized TD factor (however, this association did not survive correction for multiple comparisons). The findings of our study are consistent with qualitative accounts of the role of the self in TD (Lysaker and Lysaker, 2006) and complement what is already known about TD from a sociocognitive perspective. For example, several studies have supported the association between poor ToM and TD (Corcoran et al., 1995; Frith, 1992; Hardy-Baylé et al., 2003; Sprong et al., 2007). It is conceivable that such difficulties could be partly explained by lack of clarity of self-concept in the patient, although this will require investigation in future studies. The findings also provide a potential psychological mediator for research on the social origins of TD. For example, some studies have reported significant associations between childhood adversity (Shah et al., 2014; Toth et al., 2011) or institutionalization (Walker et al., 1981) and TD. Adoption studies have reported an interaction between genetic high-risk status and family miscommunication in the long-term prediction of TD in adoptees (Wahlberg et al., 1997, 2004). It is conceivable that clarity of self-concept could play a mediating role between these risk factors and TD.

Contrary to our expectations, the only significant between-group differences on the VISQ subscales were for other people in inner speech and condensed inner speech, with the patients scoring higher on both subscales. We did not find significant differences between the groups on self-reported dialogic inner speech or evaluative and motivational inner speech. TD, from the non-salient interview only, was associated with condensed inner speech, but this association was weak and barely significant.

A more complex picture of the relationship between inner speech and TD emerged when we considered the six TD factors. Controlling for other symptoms, we found that dialogic inner speech, other people in inner speech, and evaluative/motivational inner speech were all negatively correlated with our negative TD factor. However, when we corrected for multiple comparisons, only the association between the negative TD factor and dialogical inner speech survived. The main TD item contributing to the negative factor was poverty of speech. Hence, an implication of this finding is that the absence of social speech is correlated with a reduction in dialogical inner speech, an association that makes sense within the context of Vygotsky’s developmental model (1934), which proposes that the ontogeny of inner speech lies in social speech. The finding of an association between poverty of speech and diminished self-reported dialogical inner speech also informs the longstanding debate of whether TD is a speech or a cognitive problem (Chaika, 1982; Lanin-Kettering and Harrow, 1985).

Therefore, our findings suggest that the negative and positive dimensions of TD may be associated with different psychological processes. More specifically, negative TD/poverty of speech seems to be associated with deficits in dialogical inner speech, whereas positive TD, comprising the disorganized aspects of TD, is associated with poor clarity of self-concept.

A secondary aim of the present study was to examine the relationship between inner speech and hallucinations. Other people in inner speech was significantly correlated with hallucinations and delusions in our clinical group, as was evaluative/motivational inner speech. However, when we corrected for multiple comparisons, the only significant association was between hallucinations and other people in inner speech. Moreover, we ran partial correlations between other people in inner speech and each symptom, controlling for the other. Only the association with hallucinations remained significant. These findings are perhaps unsurprising given that auditory hallucinations take the form of the voices of others (often others who can be identified by the hearer; Nayani and David, 1996) and also given theoretical accounts that suggest that AVHs consist of inner speech that is misattributed to external sources (Bentall, 1990; Fernyhough, 2004; Frith, 1992). In this context, it is important to note that our participants were asked to report specifically on their inner speech rather than their voice-hearing experiences. Previous studies have indicated that most schizophrenia patients report being able to distinguish their inner speech from their voices, but that this discrimination is based on the sense of controlling the experience and the distinctive content of the experience (Hoffman et al., 2008; Langdon et al., 2009). It is possible, therefore, that hearing voices is associated with inner speech characterized by identities other than the self, which only becomes misattributed as alien in the presence of other factors such as controllability and content.

The finding that the patients as a whole endorsed items relating to condensed inner speech more highly than controls suggests that patients’ inner speech is predominantly condensed. Fernyhough (2004) has proposed that it is specifically expanded inner speech that is experienced as AVHs; the increase in condensed inner speech (which is the opposite of expanded inner speech) found in the patients in this study (although not specifically in association with hallucinations) might therefore be interpreted as consistent with this hypothesis. Possibly when inner speech is predominantly condensed, other kinds of inner speech (inner speech that is emotionally charged or which involves the voices of others) are especially likely to be experienced as anomalous and hence misattributed to an external source, particularly if patients also have other vulnerabilities to making these kinds of misattributions, for example impaired source monitoring (Brookwell et al., 2013).

### Limitations

As in most studies of TD, we only recruited patients with psychotic-spectrum diagnoses, but there is evidence that TD is a transdiagnostic construct, especially affecting patients with a bipolar diagnosis (Andreasen, 1979b; Tai et al., 2004). Another limitation is that we used a questionnaire to quantify inner speech. The VISQ has already been used to examine sub-syndromal psychotic experiences in healthy samples (Alderson-Day et al., 2014; McCarthy-Jones and Fernyhough, 2011). However, the methodology relies heavily on the patient’s metacognitive ability to reflect about thoughts, and this ability may be compromised in some patients (Van der Meer et al., 2010).

The same limitations apply to the use of the self-report measures to assess clarity of self-concept. Although the SCCS has been used in other studies with participants diagnosed with psychotic-spectrum disorders (*e.g.*, Evans et al., 2015), the ability to report on one’s clarity of self-concept may depend on metacognitive abilities, which are known to be compromised in patients diagnosed with psychosis (Savla et al., 2013). This kind of impairment would most likely reduce our ability to detect associations between self-concept clarity and other variables, rather than lead to spurious correlations, but it should nonetheless be born in mind when interpreting the data.

In future research, one way of circumventing the limitations of the VISQ may be to complement the methodology with Descriptive Experience Sampling (Hurlburt and Akhter, 2006). This method allows for inner experience to be captured in the moment. It would also be interesting to include more comprehensive and phenomenological way of exploring self-disturbances such as the *examination of anomalous self-experience* (Parnas et al., 2005).

### Implications for Clinical Practice

The most obvious implication relates to the therapeutic strategies adopted when working with thought-disordered patients. The findings seem to suggest that therapeutic work with patients who present with predominantly poverty of speech should focus on improving dialogical inner speech by perhaps promoting and incentivizing socialization and opportunities for the patient to converse. Therapeutic work with patients who present with predominantly positive TD and disorganization should perhaps focus more on improving self-concept through consistent and coherent feedback about patients’ self-knowledge and self-beliefs (Slotter and Gardner, 2014). This work should be carried out carefully given that interpersonal sensitivity seems to have an important moderating effect of TD (Grant and Beck, 2009). Lysaker and Lysaker (2002) suggest three main requirements for the rehabilitation of patient’s dialogical processes, namely, a non-hierarchical relationship that promotes the patient’s dialogue and self-disclosure, a commitment to helping the patient remember and explain personal views and concerns, and finally the use of strategies to promote and assist patients as they converse within themselves and with significant others about their feelings and their own representation of events.

## Figures and Tables

**APPENDIX 1:**
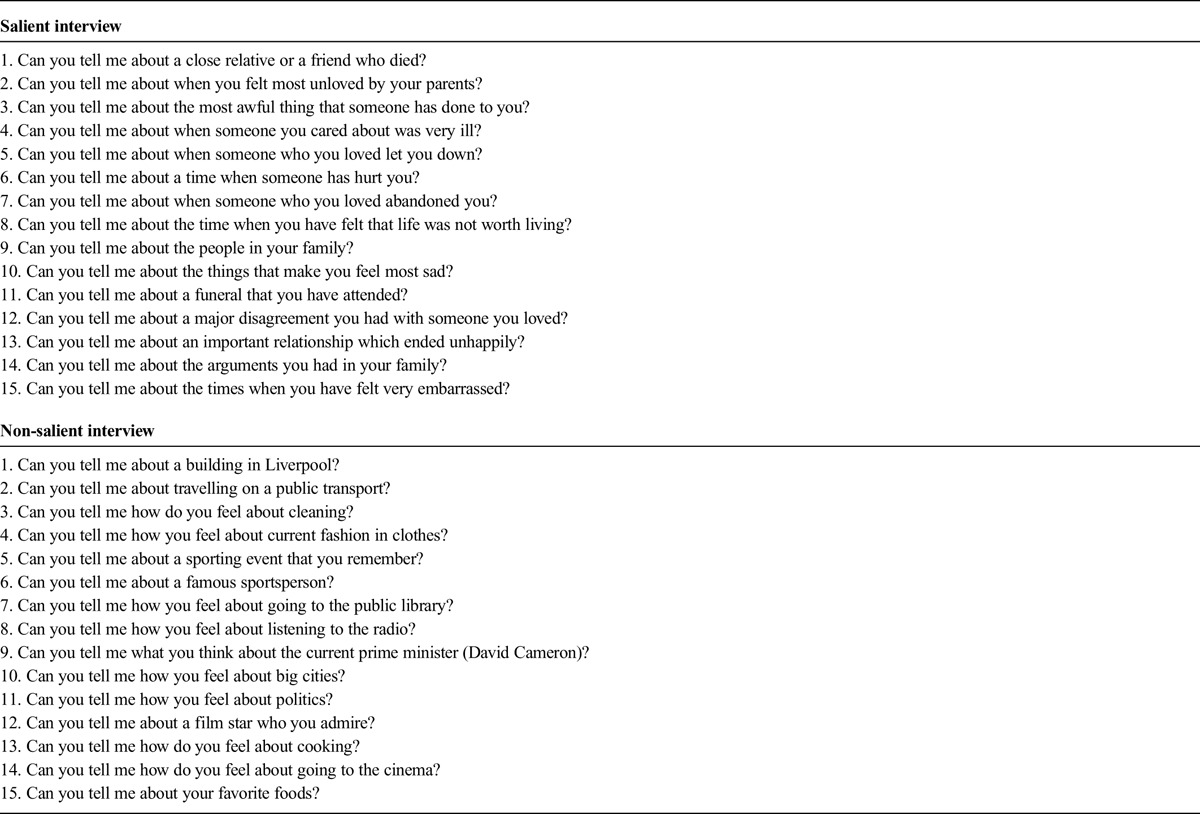
Items Used in the Salient and Non-Salient Interviews
